# Comparing the Effects of Repetitive Transcranial Magnetic Stimulation and Electroconvulsive Therapy in the Treatment of Depression: A Systematic Review and Meta-Analysis

**DOI:** 10.1155/2014/135049

**Published:** 2014-07-21

**Authors:** Beppe Micallef-Trigona

**Affiliations:** Department of Psychiatry, Mount Carmel Hospital, Attard, ATD 9033, Malta

## Abstract

Electroconvulsive therapy (ECT) is the longest standing psychiatric treatment available and has unequivocal benefit in severe depression. However this treatment comes with a number of side effects such as memory impairment. On the other hand, Repetitive Transcranial Magnetic Stimulation (rTMS) is a relatively new form of treatment which has been shown to be efficacious in patients suffering from a number of psychopathologies, including severe depression, with few reported side effects. Due to its potential therapeutic efficacy and lack of side effects, rTMS has gained traction in the treatment of depression, with a number of authors keen to see it take over from ECT. However, it is not clear whether rTMS represents a therapeutic alternative to ECT. This meta-analysis will therefore compare the “gold standard” treatment for severe depression, with the relatively new but promising rTMS. A literature search will be performed with the intention to include all randomised clinical trials. The null hypothesis is that there is no difference in the antidepressant efficacy between the two types of treatment modalities. Statistical analysis of Hamilton Depression Rating Scale (HDRS) scores will be performed.

## 1. Introduction

ECT is one of the oldest forms of treatment in psychiatry that is still used today. A recent systematic review showed “*significant superiority of ECT in all comparisons: ECT versus simulated ECT, ECT versus placebo, ECT versus antidepressants in general, ECT versus TCAs, and ECT versus MAOIs*” [1]. This confirmed the findings by the UK ECT Review Group [2] which had found that ECT is an “*effective short-term treatment for depression and is probably more effective than drug therapy.*” The consortium for Research in ECT [3] also found a strong positive effect for ECT, with remission rates of 75% after the first two weeks of use, in patients suffering from acute depressive illness. In a recent study by Kellner et al. [4] 200 patients diagnosed with unipolar depression showed better remission rates after bilateral ETC when compared to the control group on Nortriptyline and Lithium. ECT has also been shown to have positive effects in patients suffering from severe depression with psychosis, who saw a remission rate of up to 90%. However, the symptomatic remission induced by ECT appears to be short-lived [5]. ECT is also effective, especially when given bilaterally, in the manic phase of Bipolar Affective Disorder (BPAD) [6, 7]. However, given the good efficacy of current drug therapy, ECT is usually limited to patients with specific contraindications to pharmacological approaches or who are refractory to pharmacological treatment. In schizophrenia, ECT has been successfully used in both the acute phase, especially in patients with catatonic symptoms [8], as well as in chronic treatment resistant cases [9]. ECT has been used in a number of other conditions such as Obsessive Compulsive Disorder [10], Postpartum Psychosis [11], and Parkinson's disease [12], with varying degrees of success.

A British Journal of Psychiatry systematic review [13] showed “*an effect in favour of rTMS compared with sham after 2 weeks of treatment, but this was not significant at the 2-week follow-up.*” The study also found that the quality of the included clinical trials available was low and provided insufficient evidence to justify the use of rTMS in the treatment of depression. However an earlier meta-analysis, performed on 12 studies comparing HDRS score reduction with rTMS and sham stimulation, found rTMS to be statistically superior in the treatment of depression [14]. This was confirmed in a recent meta-analysis of 34 studies [15], which concluded that rTMS had a significant effect size difference of 0.55 when compared to sham stimulation in the treatment of depression. A recent presentation [16] at the American Psychiatric Association's 2013 Annual Meeting, involving a multicentre, longitudinal, naturalistic, observational study, confirmed that acute rTMS induced “*statistically and clinically meaningful response and remission*” in patients with major depressive disorder during the acute phase, but more importantly that the results were maintained at 52 weeks.

One advantage of rTMS over ECT is that the patient does not need anaesthesia, as well as the fact that seizures need not be induced. Therefore, rTMS has a much safer risk profile compared to ECT. Common side effects of rTMS are headache, twitching of the facial muscles, and auditory impairments, during the actual treatment. With respect to severe acute adverse effects, induction of seizures, though rare, has been known to occur. In the early days of rTMS accidental seizures were reported and thought to be caused by overstepping of safety limits now in place. However, in their report on the safety of rTMS, Rossi et al. conclude that the risk of rTMS to induce seizures is very low, taking into account the large amount of study data that exists from 1998 [17]. When adverse effects occur, they are usually mild and transient and rTMS is considered to be a safe therapeutic intervention [18]. With respect to adverse cognitive effects, it is well known that many psychiatric disorders, such as depression and schizophrenia, have a negative impact on cognitive functions. However, unlike ECT, rTMS has shown to improve cognitive functions, including both short as well as long term memory [19]. In fact, a 2003 study reported that rTMS patients “*demonstrated mild improvement on tests of working memory and retrograde memory and relative stability on tests of verbal learning and retention”* [20].

## 2. Materials and Methods

### 2.1. Hypothesis

This meta-analysis sets out to compare ECT to the relatively new but promising rTMS, for the management of treatment resistant depression. The null hypothesis being tested is that there is no statistically significant difference in the antidepressant efficacy between the two types of treatment modalities.

### 2.2. Literature Search

A literature search was carried out with OvidSP using the following resources: MEDLINE (1996 to January Week 5 2013), EBM Reviews—Cochrane Database of Systematic Reviews (2005 to January 2013), EBM Reviews—ACP Journal Club (1991 to January 2013), EBM Reviews—Cochrane Central Register of Controlled Trials (1991–January 2013), Embase (1974 to 2013 Week 06), and PsycINFO (1806 to February Week 1 2013). Search was carried out with a* limit* for clinical trials, using the Medical Subject Headings (MeSH) search terms* electroconvulsive therapy* AND* transcranial magnetic stimulation*. The titles of the results were examined to indicate suitability, followed by a detailed analysis of the entire study in order to ascertain whether the study met the established selection criteria. A manual search was then performed across the reference lists of the identified studies in order to determine if any additional studies could be included.

### 2.3. Selection Criteria

To be included in the meta-analysis the clinical trial should (1) compare the effect of ECT and rTMS in the treatment of depression, (2) be a randomised clinical trial (RCT) published in an English journal, (3) be conducted on human subjects who are over the age of 18 and have given informed consent, (4) be prospective with parallel design, (5) meet ICD-10 or DSM-IV criteria for unipolar depression or bipolar depression with a current depressive episode, (6) have HDRS assessed shortly before and after treatment, and (7) report HDRS score and standard deviation (SD). A single exclusion criterion was used: comorbid drug abuse.

### 2.4. Evaluation of Individual Studies

Each study was meticulously examined and evaluated for study quality. The quality of the existing literature was judged using Chalmer's method [21]. The heterogeneity of study design was also assessed.

### 2.5. Data Extraction

After the completion of the database search, the articles were read carefully and a brief summary of each article was written. The studies were then tabulated under the following headings: authors, the year of publication, the number of participants, rTMS or ECT, gender, psychotropic drugs, the type of rTMS technology used, the type of ECT technology used, and the HDRS mean and standard deviation before and after treatment in both groups.

### 2.6. Statistical Methods

Statistical analyses were done with SPSS (IBM) and Comprehensive Meta-Analysis software (Biostat). Changes in HDRS scores in participants were analysed using paired and independent *t*-tests. The effect sizes were then calculated in the 9 studies, for both rTMS and ECT. Due to the fact that the sample sizes of the studies were small,* Hedges' g* was used rather than* Cohen's d*, in order to reach a better estimate of effect size [22]. Since one study did not provide raw scores of HDRS and SD after completion of treatment, instead of the pooled standard deviation, the SD value obtained before the treatment was used. Since homogeneous variance is assumed for the effects within a group, this should have little effect on the end result [23]. An average effect size was calculated for both groups; effect sizes were summed and divided by the number of effect sizes. After the effect sizes were calculated within the groups, a homogeneity analysis was performed. This type of analysis examines whether the effect sizes differ significantly. If homogeneity investigation shows varying effect sizes, it may be justifiable to not pool together effect sizes and instead make an additional analysis in order to see if certain characteristics can differentiate the studies [24]. Three measures of heterogeneity were tested: *Q* Test, *z* test, and Radial plot (Galbraith Test). Publication bias was then ascertained using two methods: Rosenthal's File drawer Method and Rank Correlation. The final step was the analysis of combined effect sizes using the Fixed and Random Effects Models [25].

## 3. Results

### 3.1. Participants and Treatment

The meta-analysis included 9 randomised clinical trials published between 2000 and 2011 with a total of 384 participants.  Table 1 shows the patient characteristics, including the number of study participants, their mean age and the duration of depressive episode, the rTMS and ECT treatment protocols used in the studies, as well as the diagnosis. With respect to the diagnosis, participants in all studies had a current diagnosis of Major Depressive Disorder (MDD). In two studies [26, 27], this included patients with a history of Bipolar Affective Disorder (BPAD). In two other studies [20, 28], information was unclear on whether patients with a previous history of BPAD were included. Most of the participants in the studies were referred due to treatment (drug) resistance, which was in fact an actual selection criterion in two of the studies [28, 29]. Whilst no reference was made to the grade of treatment resistance in the studies, treatment resistance is a term used to describe cases of major depressive disorder that do not respond adequately to adequate courses of at least two antidepressants [30].

### 3.2. Treatment Characteristics

ECT patients varied with respect to their psychopharmacological treatment, with two studies requiring that patients be off treatment before commencement of the study [29, 34]. In two other studies [20, 28], ECT was used as an add-on to the patient's psychotropic treatment, while patients undergoing rTMS were required to withdraw medications.

### 3.3. Statistical Analysis

#### 3.3.1. Paired *t*-Test for HDRS Improvement

The average improvement in HDRS scores for both groups was substantial. The participants who underwent rTMS had a mean reduction of 9.3 points on the HDRS (SD 4.45, SE 1.48) which was found to be significant with *t* = 6.274 (*P* ≤ 0.0005 at 95% CI). ECT participants had a mean reduction of 15.42 points on the HDRS (SD 4.55, SE 1.52) which was also found to be significant with *t* = 10.18 (*P* ≤ 0.0005 at 95% CI).

#### 3.3.2. Independent *t*-Test for Difference in Groups

A two sample *t*-test (independent *t*-test) showed significant difference between the rTMS and ECT groups with respect to decrease in HDRS scores (*t* = 2.89, *P* = 0.011).

#### 3.3.3. Effect Size

Bias Corrected (Hedges) Effect Sizes was calculated in the 9 studies, for both rTMS and ECT. Effect sizes ranged from 0.56 to 2.83. The mean effect size for rTMS was 1.33 whilst that for ECT was 2.14.  Table 2 summarises these results.

#### 3.3.4. Independent *t*-Test for Difference in Groups with Effect Size

A two sample *t*-test (independent *t*-test) showed significant difference between the rTMS and ECT groups when effect size was taken into consideration (*t* = 3.61, *P* = 0.002).

#### 3.3.5. Homogeneity Analysis for rTMS Studies

**Figure 1:**
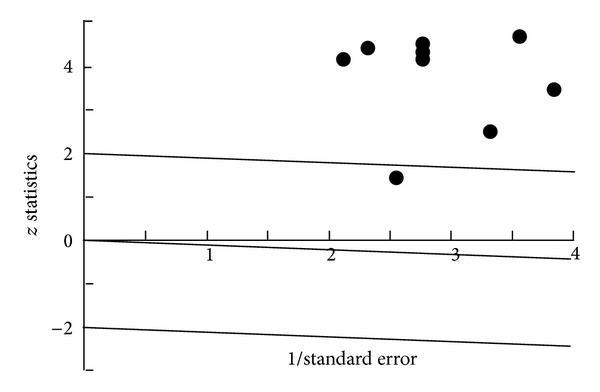
Radial Plot rTMS.

 The *Q* Test: *Q* = 14.3328  df = 8  *P* = 0.0735  *I*2 = 44.2%.
 The *z* Test: Mean *z* = 0.1349 SD *z* = 1.3308.
 Radial Plot can be seen in Figure 1.

**Figure 2:**
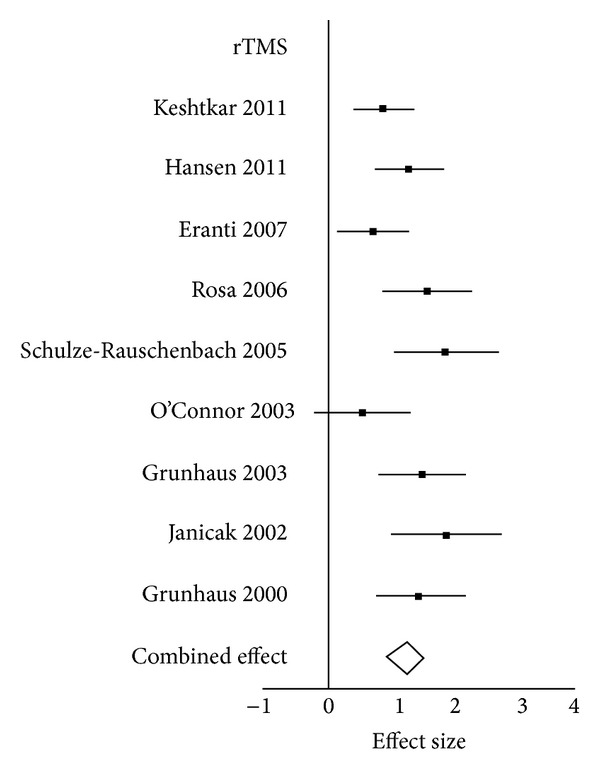
Forrest Plot rTMS.

Homogeneity analysis showed significant heterogeneity in the combined effect sizes for the rTMS studies; therefore, additional analysis was performed. An investigation was carried out to check out the validity of the data from the studies. However, no obvious reason for heterogeneity was found, and a decision was made to proceed with the Random Effect Model for combined Effect Size analysis. A forest plot can be seen in Figure 2.

#### 3.3.6. Homogeneity Analysis for ECT Studies

**Figure 3:**
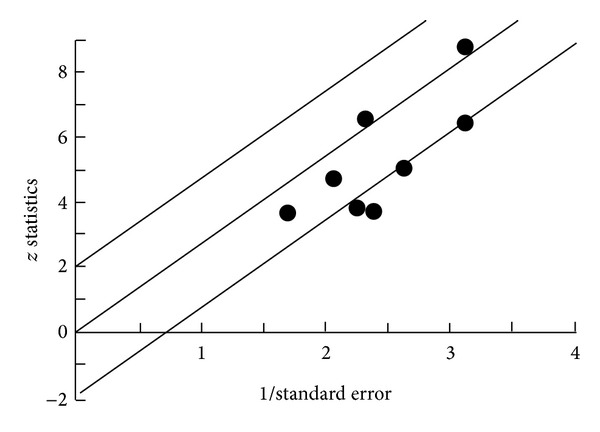
Radial Plot ECT.

 The *Q* Test: *Q* = 10.9267  df = 8  *P* = 0.2059  *I*2 = 26.8%.
 The *z* Test: Mean *z* = −0.0435 SD *z* = 1.1678.
 Radial Plot can be seen in Figure 3.

**Figure 4:**
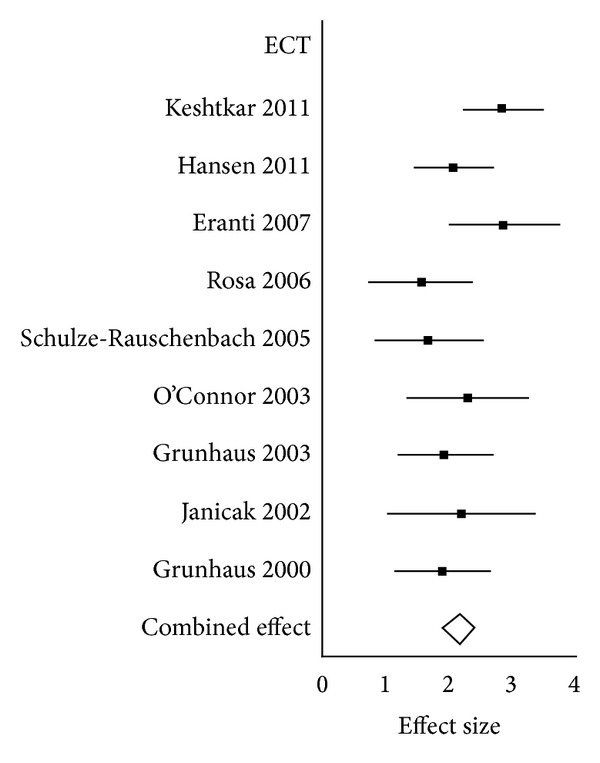
Forrest Plot ECT.

Homogeneity analysis showed no significant heterogeneity in the combined effect sizes for the ECT studies; therefore, not any additional analysis was performed. Forest plot can be seen in Figure 4.

#### 3.3.7. Combined Effect Size for rTMS and ECT Studies

Combined effect sizes for Fixed and Random Effect Models can be seen in Table 3.

#### 3.3.8. Publication Bias for rTMS Studies

 
*Rosenthal File drawer Method.*
 Tolerance minimum number = 55.
 Estimated number = 282.
 Estimated not less than minimum: publication bias not detected.
 
*Rank Correlation.*
 
*z* = 0.1043  *P* = 0.4585.
 Publication Bias for ECT Studies.
 
*Rosenthal File drawer Method.*
 Tolerance minimum number = 55.
 Estimated number = 587.
 Estimated not less than minimum: publication bias not detected.
 
*Rank Correlation.*
 
*z* = −0.1043  *P* = 0.5415.

## 4. Discussion

### 4.1. Summary

To my knowledge, this is the first meta-analysis that set out to analyse and compare ECT, the “gold standard” treatment for severe depression, with the relatively new but promising rTMS. Following extensive literature review, nine randomised controlled clinical trials were included. The null hypothesis, that is, that there is no significant difference in the antidepressant efficacy between the two types of treatment modalities, was tested using a number of statistical measures and was refuted.

### 4.2. Main Conclusions

The results of this meta-analysis show that patients who undergo either rTMS or ECT have statistically significant reductions in their depressive symptoms, as measured by HDRS. rTMS participants had a mean reduction of 9.3 points whilst ECT participants had a mean reduction of 15.42 points on the HDRS. This is an important conclusion, especially when one takes into account the fact that the majority of participants who were referred for the studies were refractory to treatment with psychotropic medication. When the degree of improvement between rTMS and ECT participants was analysed, those participants undergoing ECT showed significantly lower HDRS scores compared to those undergoing rTMS. When the effect size was factored into the comparison, the difference became even more significant in favour of ECT. This conclusion reaffirms ECT as the leading therapeutic modality for patients with treatment resistant depression, purely on the basis of depressive symptomatology outcome, but also suggests the therapeutic validity of rTMS as a treatment tool for depression in patients who are treatment resistant.

### 4.3. Individual Study Conclusions

Three of the nine studies concluded that the antidepressant effect of ECT is significantly larger than rTMS when the groups were compared (Eranti et al., 2007) [27, 28]. The other studies did not report any significant difference when both groups were compared; however, Keshtkar et al. (2011) showed that the decrease in the score for suicidal subscale score of HDRS was significantly greater in the ECT group than in the rTMS group. This meta-analysis therefore served to confirm the results of these studies. The studies which did not show significant differences between the rTMS and ECT participants (Grunhaus et al., 2003; Schulze-Rauschenbach et al., 2005) [20, 26, 29] were further analysed in order to ascertain possible causes for lack of significant difference between the groups. The first factor analysed was the age of participants, since Figiel et al. (1998), Kozel & George (2002), and Pallanti et al. [30] have reported that older patients had poorer outcome when undergoing rTMS compared to younger patients. Interestingly, in Janicak et al. [26], the authors observed that the younger patients seem to need fewer rTMS treatments to achieve the response as compared to older people. In fact, two of the three studies which showed significant difference (Eranti et al., 2007) [28] had the highest mean age for participants undergoing rTMS. However, one must be cautious before making predictive conclusions from such a correlation and further studies need to be carried out in order to confirm such findings. Another factor studied was the type of ECT that was administered to patients, since it is known that bilateral ECT has better outcomes when compared to unilateral ECT [35]. However, no correlation was found between the studies with respect to electrode placement in ECT participants. When looking at the diagnosis of participants in the studies, it can be noted that some studies had participants with only unipolar depression [28] (Grunhaus et al., 2003; Rosa et al., 2003) whilst the rest had patients with both unipolar and bipolar depression.

Whilst ECT is known to be effective in patients who are in both their depressive as well as their manic phases of their bipolar illness [36], rTMS has not been substantially studied in such patients. This raises the question of whether rTMS has a similar effect on bipolar and unipolar depression. At a similar tangent, in one of the studies [28], participants with psychotic depression showed significantly less improvement with rTMS. This observation should therefore be addressed in future studies. Interestingly, publication bias was not detected, using both the Rosenthal File drawer Method as well as Rank Correlation. This may be, in part, due to the recent efforts of prominent medical journals that require authors to register their trial before it begins. Therefore, unfavourable results are published and not withheld.

### 4.4. Limitations

#### 4.4.1. Control Groups

One of the main limitations of this meta-analysis, which reflects the limitations of the individual studies, is that placebo/sham groups were not used; although the studies were randomised and controlled, a sham comparison group was not used. As described by Grunhaus, this lack of control may constitute a problem in both rTMS as well as ECT groups:* “The placebo components of rTMS (daily contact, popular beliefs on the effects of magnets, etc.) could be powerful. Similar psychological issues, however, could be in effect in the ECT-treated patients in whom an aversion to the treatment may constitute a strong factor for an escape into health”* [28]. However, as stated by a number of the study authors, due to the severity of the depression, it was not thought to be ethically justifiable to have a placebo/sham group.

#### 4.4.2. Blinding

Another important consideration which must be taken into account is that the studies were not blinded, both with respect to observers as well as to participants. In fact, only one of the studies was single blind with respect to observers [29]. Even though patients were asked not to reveal their group allocation, the strength of this single blind remains dubious. Although the authors gave a rationale for the lack of blinding, this still remains one of the limitations present and should be addressed in future comparative studies.

#### 4.4.3. Psychotropic Medications

In three of the studies [28] (Grunhaus, et al., 2003, O'Connor et al., 2006), participants in the ECT group were allowed to continue their psychotropic medications, whilst the participants in the rTMS group were not. The contribution that psychotropic medications had on the outcome of these studies is therefore up for debate. In fact, a number of studies [37, 38] have shown that a combination treatment of rTMS and antidepressants results in a significant improvement when compared with placebo.

#### 4.4.4. Treatment Protocols

In some of the studies ECT was continued until treatment response, while rTMS was administered over a predetermined schedule. As suggested by Eranti et al. (2007)* “Although the mean durations of the rTMS and ECT courses were comparable, it could be suggested that many more weeks of rTMS were required.”* ECT dose was also adjusted secondary to participant response and not according to a fixed protocol in the majority of studies.

#### 4.4.5. Definition of Response

One of the main outcomes of the included studies was response and remission according to HDRS scale. Response is commonly defined as a reduction in HDRS score of 50% or more whilst remission as a final HDRS score of eight or less [39]. Unfortunately, the studies had varying definitions of response and remission. Hansen et al. [27], for example, use the term partial remission as their endpoint, which was equal to an HDRS score of twelve or less.

#### 4.4.6. Definition of Treatment Resistance

As previously discussed, treatment resistance generally occurs when there is inadequate response to at least two antidepressants [30]. However, none of the studies discussed the grade of treatment resistance and studies did not indicate what antidepressant treatment was tried before the patient was classified as treatment resistant.

#### 4.4.7. Limitations of Meta-Analysis

As previously discussed, study participants differed with respect to age and sex distribution, drug therapy, type of depression, psychotic symptoms, treatment parameters, and blinding. Nevertheless, all studies reported initial and endpoint HDRS scores, which was one of the main criteria for admission of the study into the meta-analysis. In addition to the limitations described above, the main limitations of the meta-analysis were that the studies that have been carried out (and included in this meta-analysis) comparing rTMS and ECT are small, with a limited number of participants (between 25 and 73) which makes generalisation of results difficult. The main reason for this is that rTMS is still in its infancy when it comes to routine clinical use, and in many places it remains an experimental technique. As the use of rTMS gains traction, further studies, with larger sample populations, will allow further investigation of the benefits of rTMS, and therefore a better comparison with ECT can take place.

#### 4.4.8. HDRS

Another limitation to consider is the use of the HDRS. A number of studies have criticised the scale's ability to measure the severity of the patient's depression and have concluded that the scale suffers from poor retest and inter-rater reliability, poor content validity, as well as poor replication across samples [40, 41].

### 4.5. Future Work

As a result of the limitations listed above, a number of improvements should be sought in future meta-analyses. However, since many of the limitations are due to the design of the studies themselves, this will add a degree of difficulty for subsequent meta-analyses to overcome. Future work on the effects of patient age and diagnosis of unipolar versus bipolar depression, when comparing the two treatment modalities, would help to shed further light onto the significant difference found in this meta-analysis. Comparison of the short term and long term side effects would also be an essential step in the risk/benefit analysis of the two treatment modalities, allowing clinicians to select the most appropriate modality for particular patients.

### 4.6. Concluding Remarks

In conclusion, this report comparing the effects of rTMS and ECT in the treatment of depression, whilst showing ECT's superiority when compared directly to rTMS, also shows that rTMS can be considered as a useful adjunct to the therapeutic interventions in depression currently available. Whilst not proving beyond reasonable doubt the efficacy of rTMS, this meta-analysis indicates an important role for rTMS in the management of treatment resistant depression, especially in select patient groups. However, the future success and deployment of this treatment modality will largely be contingent upon further technological as well as logistical advances that can help to clarify effective use, cost-effectiveness, access to treatment, and patient selection.

## Figures and Tables

**Table 1:** Patient characteristics and treatment protocols.

Study	*n*	Age		Episode duration (mo)		Treatment protocol		Diagnosis
		rTMS	ECT	rTMS	ECT	rTMS	ECT	
Keshtkar 2011 [31]	73	34.0	35.6	n.a.	n.a.	MS: 10 MT: 90% TP: 4080	MS: 10 BL: 100%	UP
Hansen et al. 2011 [27]	60	46.0	52.0	6.0	4.0	MS: 15 MT: 110% TP: 1800	MS: 9 UL: 100%	UP + BP
Eranti 2007 [32]	46	63.6	68.3	7.1	5.6	MS: 15 MT: 110% TP: 15000	MS: 6.3 UL: 18% BL: 82%	UP
Rosa et al. 2006 [29]	42	41.8	46.0	110.7	103.6	MS: 20 MT: 100% TP: 50000	MS: 12 UL: 100%	UP
Schulze-Rauschenbach et al. 2005 [33]	30	47.7	46.7	n.a.	n.a.	MS: 10.8 MT: 100% TP: 20000	MS: 9.9 UL: 100%	UP
O'Connor et al. 2003 [20]	28	51.2	48.4	n.a.	n.a.	MS: 10 MT: 90% TP: 16000	MS: 6–12 UL: 100%	n.a.
Grunhaus et al. 2003 [34]	40	57.6	61.4	16.6	10.4	MS: 20 MT: 90% TP: 24000	MS: 10 UL: 65% BL: 35%	UP
Janicak et al. 2002 [26]	25	42.9	42.7	5.5	3.1	MS: 20 MT: 110% TP: 20000	MS: 12 BL: 100%	UP + BP
Grunhaus et al. 2000 [28]	40	58.4	63.6	8.3	6.9	MS: 20 MT: 90%. TP: 8000–24000	MS: 9.6 UL: 60% BL: 40%	n.a.

Notes

*n* = number of participants; n.a. = data not available; UP = unipolar depression; BP = bipolar depression; MS = mean number of sessions; MT = motor threshold; TP = total pulses; UL = unilateral ECT; BL = bilateral ECT.

**Table 2:** Bias corrected (Hedges) effect sizes for each study.

	rTMS				ECT			
Study			Confidence interval				Confidence interval	
	Effect size	Standard error	Lower	Upper	Effect size	Standard error	Lower	Upper
Keshtkar 2011 [31]	**0.90**	0.26	0.39	1.40	**2.82**	0.32	2.21	3.44
Hansen et al. 2011 [27]	**1.31**	0.28	0.75	1.87	**2.07**	0.32	1.45	2.70
Eranti 2007 [32]	**0.74**	0.30	0.16	1.33	**2.83**	0.43	1.99	3.66
Rosa et al. 2006 [29]	**1.61**	0.36	0.89	2.32	**1.56**	0.42	0.74	2.38
Schulze-Rauschenbach et al. 2005 [33]	**1.90**	0.43	1.07	2.73	**1.67**	0.44	0.81	2.53
O'Connor et al. 2003 [20]	**0.56**	0.39	−0.20	1.31	**2.27**	0.48	1.32	3.22
Grunhaus et al. 2003 [34]	**1.54**	0.36	0.83	2.25	**1.93**	0.38	1.18	2.68
Janicak et al. 2002 [26]	**1.93**	0.47	1.00	2.86	**2.17**	0.59	1.00	3.33
Grunhaus et al. 2000 [28]	**1.49**	0.36	0.79	2.19	**1.90**	0.38	1.16	2.65

**Table 3:** Combined effect sizes for fixed and random effect models.

	rTMS				ECT			
Model			Confidence interval				Confidence interval	
	Effect size	Standard error	Lower	Upper	Effect size	Standard error	Lower	Upper
Fixed effect	1.24	0.11	1.01	1.46	**2.17**	**0.13**	**1.90**	**2.43**
Random effect	**1.28**	**0.15**	**0.97**	**1.58**	2.15	0.16	1.85	2.46
